# Supplementary material for: E7050 Suppresses the Growth of Multidrug-Resistant Human Uterine Sarcoma by Inhibiting Angiogenesis via Targeting of VEGFR2-Mediated Signaling Pathways
Source: Int J Mol Sci. 2023 May 31;24(11):9606. doi: 10.3390/ijms24119606 (PMC10253979; doi:10.3390/ijms24119606)
Supplement: Supplementary file 1 [file ijms-24-09606-s001.zip › ijms-2340065-supplementary.pdf]

Supplementary Materials

E7050 Suppresses the Growth of Multidrug-Resistant Human Uterine Sarcoma by Inhibiting Angiogenesis via Targeting the VEGFR2-Mediated Signaling Pathways

Tsung-Teng Huang, Chuan-Mu Chen, Ching-Chiung Wang, Song-Shu Lin, Ying-Wei Lan, Hsu-Chen Cheng, Kong-Bung Choo, Tse-Hung Huang and Kowit-Yu Chong

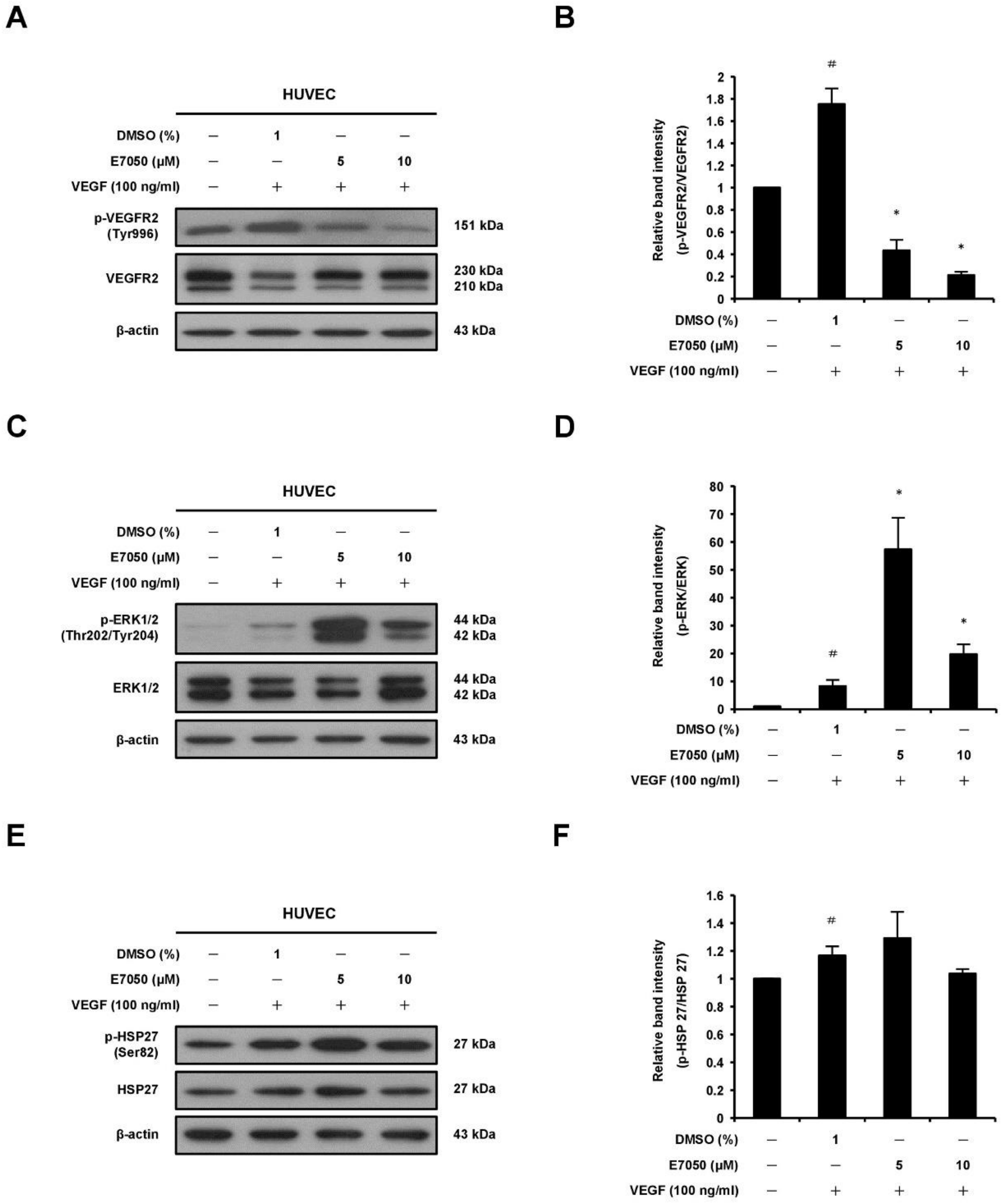

Figure S1. Effects of E7050 on the expression of p-VEGFR2 (Tyr996), p-ERK1/2 (Thr202/Tyr204) and p-HSP27 (Ser82) in VEGF-stimulated HUVECs. Cells were serum-starved for 6 h, pretreated with E7050 (5 and 10 μM) or vehicle for 1 h, followed by the stimulation with VEGF (100 ng/ml) for another 10 min (VEGFR2) or 30 min (ERK1/2 and HSP27). Total cellular proteins were extracted and Western blotting was performed. (**A**) E7050 significantly inhibited the phosphorylation of VEGFR2 at Tyr996 in a concentration-dependent manner. (**B**) Quantification of p-VEGFR2 levels after normalizing to total VEGFR2 protein levels. (**C**) Effects of E7050 on the protein levels of total ERK1/2 and p-ERK1/2 in VEGF-stimulated HUVECs. (**D**) Quantification of p-ERK1/2 levels after normalizing to total ERK1/2 protein levels. (**E**)

Effects of E7050 on the protein levels of total HSP27 and p-HSP27 in VEGF-stimulated HUVECs. (**F**) Quantification of p-HSP27 levels after normalizing to total HSP27 protein levels. The data are presented as mean ± SEM of three independent experiments. $^{\#}P < 0.05$ compared with the untreated cells. $^{*}P < 0.05$ compared with the vehicle-treated cells.
